# Living With Chronic Lower Pulmonary Disease: Disruptions of the Embodied Phenomenological Self

**DOI:** 10.1177/2333393614548762

**Published:** 2014-10-08

**Authors:** Charlotte Pooler

**Affiliations:** 1 University of Alberta, Edmonton, Alberta, Canada

**Keywords:** asthma, chronic obstructive pulmonary disease (COPD), dyspnea, embodiment / bodily experiences, illness and disease, chronic, lived experience, Merleau-Ponty, phenomenology, respiratory disorders, van Manen

## Abstract

In this article, I present a phenomenological study of individuals’ experiences of living with moderate to very severe chronic lower pulmonary disease (chronic obstructive pulmonary disease, asthma, or both). Phenomenology is a philosophy, distinct from descriptive or thematic research, which is useful as a foundation for scientific inquiry. In this study, I used the lens of Merleau-Ponty to understand and interpret participants’ experiences of living with pulmonary disease, and the approach of van Manen for analysis. I conclude that in chronic pulmonary disease, awareness of breathing and the body is experienced in the sounds, sensations, and signals of breathing and the body, and in the experiences of the body-in-the-world. Central themes of being-in-the-world from the study describe the disruption of the embodied phenomenological self: Participants experienced slowing down, doing less, and having to stop due to shortness of breath. Both chronic and acute dyspnea were prevalent and the taken-for-granted aspects of daily activities were disrupted. Findings of this study have implications for public and patient education, and opportunities for integration of experiential aspects within nursing education and practice.

Chronic lower pulmonary disease, which includes chronic obstructive pulmonary disease (COPD) and asthma, is prevalent in most countries and poses significant challenges to both life expectancy and quality of life (Akinbami & Liu, 2011; Global Initiative for Chronic Obstructive Lung Disease [GOLD], 2013; To et al., 2012). COPD is a leading cause of death in many countries, including North America, and has significant costs and burden to both individuals and society (GOLD, 2013). Yet, current statistics underestimate the significance of COPD in that it is underdiagnosed and there are multimorbidities that occur with moderate to very severe COPD (GOLD, 2013; Vanfleteren et al., 2013). Asthma is most often identified at an earlier onset, whereas COPD is usually advanced when diagnosed. In adults, asthma is also associated with disability, poor quality of life, loss of work, and increased use of health care resources (To et al., 2012).

Persons with chronic lower pulmonary illness often experience difficulty with breathing, shortness of breath, or breathlessness, which are labeled as dyspnea. In 1999, the American Thoracic Society (ATS) issued its first consensus statement on dyspnea and defined it as “a subjective experience of breathing discomfort that consists of qualitatively distinct sensations that vary in intensity” (p. 322). In the recent ATS update, Parshall et al. (2012) noted that dyspnea is a complex symptom and “emphasize[d] strongly that dyspnea per se can only be perceived by the person experiencing it. Perception entails conscious recognition and interpretation of sensory stimuli and their meaning” (p. 437).

For sensitive, thoughtful, and effective nursing care, it is important to define, delineate, and describe concepts and symptoms, and to gain rich descriptions of patients’ experiences and their unique perceptions and meanings (Morse, 1996; Morse & Johnson, 1991). However, research into symptoms and experiences of persons living with COPD or asthma has challenges, of which perhaps the most significant is that dyspnea is a subjective symptom difficult to describe. Unlike other symptoms, such as pain, nausea, or fatigue, many persons who experience breathing symptoms do not use, and might not even recognize, the label dyspnea. Furthermore, although dyspnea is prevalent and distressing in COPD, the disease might be stigmatized, and people might conceal their symptoms (Bailey, 2004; Barnett, 2005; Boyles, Bailey, & Mossey, 2011; Clancy, Hallet, & Caress, 2009).

Over the past decade, experiences, perceptions, meanings, and interpretations have been explored in qualitative research of persons with chronic pulmonary disease, primarily with COPD. Panic, fear, anxiety, distress, and frustration are often experienced with dyspnea (Bailey, 2004; Barnett, 2005; Clancy et al., 2009; Ek, Sahlberg-Blom, Andershed, & Ternestedt, 2011; Gardiner et al., 2009; Hall, Legault, & Côté, 2010; Hasson et al., 2008). Physical limitations are frustrating, unpredictable, and distressing (Bailey, 2004; Barnett, 2005; Boyles et al., 2011; Clancy et al., 2009; Ek et al., 2011; Elofsson & Öhlén, 2004; Fraser, Kee, & Minick, 2006; Gullick & Stainton, 2008; Habraken, Pols, Bindels, & Willems, 2008; Jonsdottir, 1998; Williams, Bruton, Ellis-Hill, & McPherson, 2007). Social isolation and being homebound are common, particularly in winter of northern climates (Ek et al., 2011; Habraken et al., 2008; Hasson et al., 2008). These findings have contributed to our understanding, but less is known about day-to-day lived experiences and meanings of persons living with chronic lower pulmonary illness.

## Philosophical Approach

Phenomenology is a useful foundation for scientific inquiry into lived experiences and phenomena. Husserl, an Austrian philosopher, developed phenomenology in the early 1900s to approach, understand, and describe human experiences and phenomena (Zahavi, 2003). Phenomenology, as conceptualized by Husserl (1900/2001), is a systemic and logical description of objects, concepts, and phenomena: “This phenomenology must bring to pure expression, must describe in terms of their essential concepts and their governing formulae of themes” (p. 86). Description is furthered through intentional orientation of awareness to the phenomena of interest.

Through description and reflection on experiences and phenomena, phenomenological analysis explicates meanings and reveals themes (Husserl, 1900/2001; Merleau-Ponty, 1945/1962; van Manen, 1997; Zahavi, 2003). A phenomenological approach might be used to study human experiences and phenomena. Aims of phenomenological research include the following: to uncover, reveal, and explore descriptors; to appraise assumptions and clarify meanings; and to increase sensitivity to such experiences (van Manen, 1997).

Phenomenology is used today as an approach and foundation for inquiry, although phenomenological philosophers—such as Husserl (1900/2001), Heidegger (1927/2010), Levinas (1981), and Merleau-Ponty (1945/1962, 1964)—did not provide methods or procedures for research (Dowling & Cooney, 2012; Giorgi, 1997, 2008). Therefore, in phenomenological research, it is important to differentiate philosophers from approaches for analysis, such as those developed by Benner (1994), Giorgi (2012), and van Manen (1997). Yet, in nursing and other health sciences, philosophical perspectives are often not incorporated into research, findings are reported without description of the foundational philosophy, and philosophers are equated with selected methods or analysis (Barnett, 2005; Dowling & Cooney, 2012; Earle, 2010). Moreover, descriptive studies are sometimes described as phenomenological research without incorporation of particular philosophical perspectives and phenomenological foundations (Dowling & Cooney, 2012; Earle, 2010; Lopez & Willis, 2004; Porter, 1998; Pratt, 2012).

It has been argued that inclusion of the philosophical perspective strengthens the depth and richness of phenomenological study (Annells, 1996; Koch, 1995; Thomas, 2005; Wilde, 2003), but the distinct and sometimes competing phenomenological philosophies ought to be recognized and selected accordingly. For example, the philosophical perspective of Heidegger (1927/2010) provides insight into existential aspects of being, of Levinas (1981) into ethics and relationships, and of Merleau-Ponty (1945/1962, 1964) into the embodied life world. The philosophical lens that is selected might further depend upon whether the study focuses on lived experiences or a phenomenon (Giorgi, 2012), and whether the analysis is thematic and descriptive or extends to interpretation (Pratt, 2012; Wojnar & Swanson, 2007).

## Method

In our roles as health care providers, educators, and researchers, nurses are concerned with experiences of health and illness (Colaizzi, 1975). In this phenomenological study, I used philosophical perspectives of Merleau-Ponty (1945/1962, 1964), whose description of being-in-the-world lends well to understanding bodily aspects of an illness (Gullick & Stainton, 2008; Thomas, 2005; van Manen, 1999). Merleau-Ponty, a French philosopher and student of Husserl, described the familiar acceptance of the body as the pre-objective and pre-reflective manner of being-in-the-world. This familiar acceptance of the body is described as the embodied and phenomenological self, which is the way one lives through and in the body. Consequently, we are able to take our body for granted and engage our attention toward our activities, interactions, projects, and others (Merleau-Ponty, 1945/1962). Merleau-Ponty also provided insight into experiences of living with disruptions in the familiar acceptance of the body. Injury or illness might create moments of awareness of that which is normally taken-for-granted. In such moments, the body and self are reflected upon and become objects of awareness (Merleau-Ponty, 1945/1962, 1964).

There were two purposes of this study: first, to extend knowledge of the experiences of adults living with chronic pulmonary illness, including their everyday meanings and activities; and second, to increase understanding of, and connections between, individuals’ experiences and health professionals’ practices (van Manen, 1997, 1998, 1999). The analytical approach I used was van Manen’s (1997, 1999) method of attentive thoughtfulness, wondering, conversing, reflecting, writing, and rewriting to clarify meanings and make explicit understandings of individuals’ experiences. These nonlinear and interactive activities engaged me in the phenomenological approach to the following research question:

**Research Question:** What is it like to experience moderate to severe COPD or asthma?

I used perspectives developed by Merleau-Ponty as a lens to provide focus, attention, and contrast to reveal assumptions, develop insights, grasp meanings, and delineate themes. I articulated themes through reflection and reduction, with ongoing awareness of my assumptions and attentiveness to participant descriptions. I linked the analysis and writing to concrete experiences, including the use of participant phrases for the themes.

Ethical review and approval were obtained from the University of Alberta Health Ethics Review Board. Individuals with moderate to very severe COPD or asthma were recruited from three pulmonary outpatient clinics and programs of a large metropolitan city in Western Canada. Registered nurses informed potential participants of the study and provided them with contact information. Participants then contacted me to seek further information and express their interest in participating. Consent to participate was obtained prior to beginning each interview. The interviews were conducted at the participants’ homes or at the International Institute for Qualitative Methodology, University of Alberta, Edmonton, Canada. Taxi fare or parking was paid for those who chose to be interviewed at the university. Most participants chose to be interviewed in their homes, stating it was due to fatigue or restrictions in mobility.

I conducted 18 interviews with 16 participants; 2 participants were interviewed a second time. There were 6 females and 10 males, aged 35 to 77 years. Seven participants had moderate to very severe COPD, 6 had moderate to severe episodes of asthma, and 3 had a combination of chronic pulmonary disease (COPD and asthma, COPD and bronchiectasis, or asthma and pulmonary fibrosis). The 6 participants with asthma were between 35 and 60 years old; the participants with COPD were between 50 and 77 years old. Disease severity was determined by pulmonary function tests previously conducted by the participant’s pulmonary specialist. Consistent with the overlap and progressive nature of chronic pulmonary illness, several participants had additional comorbidities, most commonly chronic heart disease. All participants diagnosed with COPD had a history of smoking and were on disability, were unemployed, or had taken early retirement due to physical limitations. One of the participants with COPD was waiting for a lung transplant. Five participants were on long-term home oxygen therapy.

Descriptions of participant experiences were obtained through open-ended, conversational interviews, which were audiotaped and transcribed for analysis. Additional text for reflection and analysis included close-observation of participants’ behaviors and their expressions of language, autobiographies, the research literature, and my own clinical experiences as a registered nurse. Close-observation of participants’ behaviors was important because breathing is usually experienced bodily, subjectively, and holistically without awareness but with meanings embodied in gestures and actions that might go unnoticed. Although participants described many of their experiences in detail, several stated it was difficult to describe their breathing, including shortness of breath.

These explorations of living with COPD or asthma through concrete, lived descriptions enabled me to “rediscover phenomena” through exploring the layers of lived experiences (Merleau-Ponty, 1945/1962, p. 66), which then revealed characteristics and the context of living with chronic lower pulmonary illness. The conversation, research question, and ongoing analysis were used concurrently to guide questions in the first and subsequent participant interviews. Data collection and analysis overlapped, with analysis beginning after the first interview. Collection of data was discontinued when no new themes or ideas emerged from the interviews and there was sufficient data for a full and rich description of experiences.

In the phenomenological study, I linked Merleau-Ponty’s (1945/1962, 1964) perspectives of body-awareness and subjectivity-objectivity to describe experiences of living with chronic pulmonary disease: sounds, sensations, and signals; experiences of breathing; expressions of breathing; and experiencing the body-in-the-world. In this article, I present my findings on the three central themes of how persons with moderate to very severe chronic lower pulmonary disease experience the body-in-the-world.

## Experiencing the Body-in-the-World

Merleau-Ponty (1945/1962) described the body as “the vehicle of being-in-the-world” (p. 94): as embodied-subjects “we are in the world through our body” (p. 239). When we experience events or participate in activities, there is an outward view of the self in looking both toward and within the world. That is, we are not continually self-reflective of our body as an object; instead, our attention is toward the world and others. Taken-for-granted, the physical body is the means of the self being-in-the-world. Moment-to-moment, we live in subject–object awareness of the body, although this is not a dichotomy or “two mutually external terms” of subject and object (Merleau-Ponty, 1945/1962, p. 102). Instead, in our day-to-day moments and experiences, the phenomenological, subjective body and the physical, reflected-upon, objective body inhabit space and time in the now with a “fusion of soul and body” (Merleau-Ponty, 1945/1962, p. 97).

In contrast, participants in this study who had moderate to severe COPD or asthma described frequent disruptions to the natural momentum of body and life experiences. Their movements were limited by breathing and the body. At times, they did not experience a simultaneous fusion of events, but discord between body and intentions. For them, the embodied phenomenological self was disrupted into an awareness of the physical self, reflection upon breathing and the body-in-the-world, and attentiveness to their activities and limitations. The alterations in tempo and actions are described in three central themes of experiencing the body-in-the-world: slowing down, doing less, and have to stop.

### Slowing Down

For participants, it was not always possible to increase the rate and depth of breathing to meet the oxygen needs of the body. Unable to speed up their breathing, body and self instead had to slow down. Participants found themselves slower than they used to be, slower than others, and out of tempo with others. Aspects of slowing down were more predominant for participants with moderate-severe to very severe COPD, although they were also revealed in descriptions of experiences of participants living with asthma. Subthemes are as follows: can’t keep up, slow it down, and move too fast.

#### Can’t keep up

Slowing down was noticed, reflected upon, and interpreted by participants over time and with different activities. Some participants noticed that they had to slow down even before their pulmonary disease was diagnosed or known. For example, one participant and his wife both noticed “how slow” he was when holidaying. In his description of events, subjective recognition of being slow moved into objective reflection upon his body and breathing:

I was swimming and snorkeling. Doing pretty good. But in comparison to my wife, I was twice as slow. She couldn’t believe how slow I was. You don’t forget how to swim. Or if you’ve snorkeled before you don’t forget how to flip your feet and stuff like that. But I wasn’t traveling. I’d look ahead and there she is way ahead of me. I got to thinking, “Well I’m not struggling for breath under here. I’m breathing what I thought was normal. But obviously I’m not keeping up to a normal person.” Things kind of hit you in the side of the head like that.

Although swimming in the warm ocean was not a familiar activity or context for this participant, his physical sensations did not lead him to perceive or interpret his body movement as unusual. He was attentive to his breathing, which seemed normal, and only noticed how slow he was when he compared his speed with that of his wife.

Several participants noticed that they were slowing down, were short of breath, or easily tired before they were diagnosed with COPD or asthma. However, they attributed other commonplace or lifestyle reasons to these perceptions, such as this participant with moderate asthma and COPD: “I’d be hiking or something and I noticed I was short of breath. But I didn’t attribute it to anything because I smoked then and figured that was the culprit.” A few thought it was because they were getting older, as did this participant with severe COPD who was diagnosed at 60 years of age: “All of a sudden, I just thought I was getting old. I didn’t think I was sick. It never occurred to me that my lungs were shot.” Another participant with severe COPD described his perception when he was slowing down:

I was doing a lot of slowing down, but I was getting up in age. I would have been forty-seven years old. So you don’t run as fast as you used to or you don’t push the lawnmower as fast as you used to.

#### Slow it down

Once these changes in breathing or movement were known within the context of illness, participants recognized that their bodies were not able to keep up with others, despite will or effort. Due to their lung disease, they needed to slow down. However, other people did not always recognize this change, and participants needed to be explicit at times when others did not notice. Some participants told others to go ahead: “My daughter would say, ‘Come on dad,’ and I said, ‘You go at your own pace. I can’t walk as fast as you guys can.’” Other participants asked others to slow down to their speed:

I’ll start walking at the same speed as the people I’m with are walking. And I can’t sustain it. They don’t notice that I’m in trouble. And I have to say to them, “I’m sorry, but you’re going to have to slow down because I can’t walk at that speed.”

The pace for persons with moderate to severe chronic pulmonary illness was much slower than the busy, quick pace of everyday life as they used to experience it. Participants slowed down by purposefully attending to the speed of the body and the timing of activities. Pacing of activities and breathing became conscious, reflective, and objective actions. Because they could not quicken their breathing rate, they often avoided activities or did them at a “far different speed.” One participant with severe asthma described, “Now, I just go slowly, take my time. I have to modify my activities and rest.” Moving slowly and taking time to rest required more time to complete activities, as described by this participant with COPD:

If I’m doing something in the house it takes me quite a while. I just go slow. When I get tired I just sit down for a minute. Two minutes. Get up and get at it again, as soon as I catch my breath. I take a long time to do it!

It was not only walking and other physical activities that had to be slowed down. Several participants described that they had to slow down speech or even stop conversations. For example, one participant explained, “I run out of breath when I talk so I stop.” Another participant described,

There aren’t many conversations that I can carry on in a normal fashion. Where I’m not conscious of the fact that I’m pacing a conversation. It seems to be more when I’m talking on the phone. I’ll just say to somebody, “Gee, you know, I’m running out of air. You start talking and talk for a while. Or let’s hang up and talk later, after I get my breath back.”

A few participants were unable to exert themselves at any speed, such as this man with severe COPD. He described that although it was difficult to accept at first, he is now “slowed down:”

I had gone into a very, very deep depression. [Coughs] I think it was a case of me sitting around thinking, “I’ve had such a terrific life. [Pause] And if this emphysema thing is going to slow me down like this. And I’m going to have to give up this and give up that,” I thought to myself [Coughs], “What’s the hell use in hanging around, with that kind of incapacity? I might as well, you know, get on the journey that is still to be run as it were, and let’s get on with it.” That’s the way I used to live. Slowly I came out of that very black pit. [Coughs] But I slow down to a very slow crawl now.

#### Move too fast

Several participants described that they forgot to slow down at times. I observed these moments in a few interviews. For example, near the beginning of one interview, the participant bent down, picked up his cat, took two steps, then stopped for a moment and breathed deeply. He then walked the remaining four or five steps to the chair at a slower pace, where he sat for several minutes and collected his breath by pursed lip breathing. In the interview, he described that moment and said, “Everything a person like me does, I have to do very slow. And I’ve never been one to be slow. Picking up that cat and bringing him back, I started rushing. And the minute I do that, I’m finished.”

Another participant got out of his chair in his own home and started to walk the six or seven steps across the room. After three steps he stopped. Breathing loudly, he stood in the middle of the room for several moments, not able to speak or move. Later in the interview, he commented that he had “gotten up and moved too fast.” These moments of forgetting to slow down were described as going “fast,” “rushing,” and “speeding.” One participant described his usual pace as starting out at a “speed walk” until he tired out and had to stop:

I’ve not learned to pace myself. I think that’s a big part of my problem. When I get out there to go walking, I speed walk. Well, speed walk for me. I go too fast the first length. And then I tire myself out and I have to do those little stops in-between.

### Doing Less

Living day-to-day with chronic pulmonary disease, participants experienced numerous limitations in their actions and interactions with others. There were frequent disruptions in their ease of mobility, habits of being-in-the-world, and spontaneity of movement. Participants described how their activities were limited and how they were required to do less. Subthemes of doing less are diminished capacity, conserving energy, and a static kind of life.

#### Diminished capacity

When describing their ways of being-in-the-world and doing less, several participants used pulmonary terminology, such as vital capacity, obstruct, restrict, and reserve. However, rather than use these terms in relation to alterations in lung function or volumes, participants identified they had less capacity for activities. For example, this participant spoke of his limitations with severe COPD:

Not being able to breathe and do the things you want to do is hell. Ah, it restricts. My capacity is compromised, and I can’t do a lot of things ’cause I don’t have volume. It’s reduced my ability to do anything to virtually zero. To heck with cutting the grass. Even putting the hose out on the lawn, I have to wrestle with it and it leaves me short of breath. It’s amazing what I can’t do anymore. It’s extremely limiting.

In phenomenological terms, participants experienced limitations, restrictions, and obstructions of the self and way of being-in the world. Situated in both a physical and social world, the limitations brought on by their pulmonary illness enveloped the participants’ family, friends, and social relationships. In other words, not only the *I* of self was restricted but also the *we* of self and others. One participant with COPD described limitations due to fatigue, unpredictable shortness of breath, and incessant coughing, and connected these limitations to his social life. For this participant, the increased severity of pulmonary disease disrupted his self in both place and time, altering his physical world of possibilities and distance:

The social side of my life is practically nonexistent. I hardly ever go out anymore for social occasions. I will go if it’s something of some momentous and of importance to me. In the old days, I’d go to a party three times a week. I very rarely go out at night now. When my wife and I go the theater, we go to matinees. When we go to the movies, we go to matinees.

The prevalence of diminished capacity due to physical limitations was evident in participants’ language: They described distances in terms of steps, stairs, or blocks. Many participants purposefully limited their activities to these short distances. For example, one participant described, “What I usually do is try and restrict it to two blocks. Going down the street. Just going around the block.”

Situated in a northern climate, the participants’ horizons were further constrained by seasons: Some had seasonal allergies, most found winter challenging, and some stayed inside for several months due to the snow and cold. For example, one participant with COPD had only been out of the house 7 times between January and May. He explained, “I haven’t been for walks because of the weather. I can’t get out in winter.” Another participant with severe asthma described that when he ventured out in the cold he struggled to walk two blocks: “It’s hard to breathe when it’s cold. Last winter, I went to Safeway and it wasn’t that cold. But it was cold enough. And this last block coming home, it was just like crawling on my hands and knees.” Another participant with severe asthma did not walk in winter at all. At the time of the interview it was spring, and she pointed out her current walk and compared it with that of the previous summer: “I usually walk that parking lot right down to the back and right back up this way. When it’s nice in the summer, I used to walk four blocks.”

#### Conserving energy

Decreased capacity of participants’ lungs limited their mobility and changed their way of being-in-the-world. They described actions and activities as “using energy” and “needing air,” whereas doing less was a strategy to “save energy” and “minimize fatigue.” In these moments, rather than experiencing the world within the phenomenological body, the participants’ focus of attention was directed to the objective physical self and conserving energy through planned activities:

I know there are things that I can do to combat it. I don’t go out on a five-mile walk and I’m not running around like a madman. I conserve my energy and I only use physical energy when it is essential that I do so.

Most participants had developed new habits, made modifications to everyday life, and planned activities to save energy. For example, three participants described strategies to decrease experiences of shortness of breath and maintain their activities:

When I go to bed at night I always go in the bathroom. And I take off my pants. And I have a hook right beside the bed. And I walk in the bedroom and I hang them up there. Every morning when I get up I make a habit just to do the reverse.

I’ve got my greenhouse set up out there. I can sit on the rail so I can dig in the ground. I just sit down and use one of those little hand trowels. And that’s what I do if I’m doing any digging. That’s the only way I can do it. And I’ve got a little hand pump watering unit out there to water a few tomatoes.

I’m no longer buying shoes with laces. I buy slip-on shoes. And I buy those nice long shoehorns so that I don’t have to bend over to put my shoes on. Because bending over and standing up I gotta take five minutes after that to catch my breath again.

Participants with asthma also described how they had to save energy, oxygen, and air, particularly in the acute period of an asthmatic attack. One participant with severe asthma stated that she would not speak during an acute event because every word required energy. She explained that she has to say less as she has less air: “When I’m that bad with the asthma, it’s less energy and less air I have to talk. The shortness of breath. To breathe, the tightness. I would point. Every word I can save, I save air.”

#### A static kind of life

In moderate to very severe chronic pulmonary disease, participants experienced doing less moment-by-moment in day-to-day activities. Motions and actions were limited by the body, which several participants described as not being able to breathe. For those with moderate to severe asthma, doing less included avoiding triggers, preventing attacks, and altering activities to minimize distressing sensations. One participant with severe asthma explained,

I try to have a protective environment. Tonight I’m supposed to go to a book club, but she has dogs, and they’ll bother me, and I’ll know that I’ll come home and not be able to sleep, and it’s not worth the struggle to breathe and that shaking. I can get out of breath going up stairs. I no longer run for buses in cold weather. It’s not necessarily the heavy breathing that worries me. It’s the pain in the lungs afterwards.

Participants’ activities were changed by their dyspnea and disease in both the present and future. Several participants described wanting to do more, but were unable to do so because of their severe shortness of breath:

I do get short of breath. It is frustrating. I’m the type of person, I just want to go, go go. Like, just let me go! And I’ve got something holding me back. It’s kind of like I’m not same person I was.

It’s purely the breathing. If I could breathe easily and properly, everything else would fall into place. The frustrating thing is not being able to get on with whatever it is I’m supposed to be doing. I just can’t do it anymore. I just can’t do it. I just can’t do it.

It really, really, really bothers me because it doesn’t allow me to participate in a lot of normal things. I’d love to work. I lose touch with a lot of things. I lose touch with communication and people. It’s no fun sitting here alone for the better part of the workday week.

I’m practically at the point where I really can’t do anything too much physically. The desire to do it is there. The will to do it is there. There are things I’d love to be doing. But the moment I start doing it my lungs protest. And I am fairly immediately short of breath. And I have to sit down and get back to normal breathing. All there is in life now is going to the movies or watching TV or reading books. It is going to have to be a static kind of life. I’ve been a physical participant in a hell of a lot of things. None of which are available to me anymore. I am much more static than I’d like to be.

Participants reflected upon desires and activities that were “lost” and made comparisons with what they used to do and would like to do. For example, one participant with COPD stated, “I feel angry quite often. I like to golf and I couldn’t golf. I used to curl and can’t do that anymore.” Another described being held back: “There’s so much stuff I would like to be doing in the house and I just can’t do it. I would never let things go the way I do now. I can’t do it. And I do feel useless.”

Compelled to do less, yet not having diminished desire, willingness, or interest, was frustrating for many participants. Two participants described living with COPD as “hell.” One said,

I’ve got a computer in the back room there and I play on the computer just about every morning. When I’m on the computer and just do nothing, I’m fine. I want to do something and I start to do it and my body just says, “No, you can’t do it.” It’s hell. I sit around. What am I gonna do?

In contrast to the many participants who expressed anger and frustration with their static and sedentary life, only one participant expressed that she no longer got frustrated. She had a long history of asthma, now compounded by COPD, and had been on supplemental oxygen for several years. She stated,

I used to get a little frustrated years ago. But not anymore. I just put up with it. It used to bother me at one time but not anymore. I suppose if I got a real bad attack I might get a little upset about it. But the way it is now, I’ve had it so long it, it’s grown on me. It’s part of me. As I would say, “It’s part of me. I put up with it.”

### Have to Stop

All of the participants described moments when they had to stop, usually to “catch their breath.” There are two subthemes within the central theme of have to stop: all of a sudden and standstill.

#### All of a sudden

Participants described experiences when they suddenly had to stop the activity that they were doing, such as talking or walking. Instances of having to stop were also observed in the interviews. In these moments, being and moving was suddenly interrupted for the participant without smooth transition to the next moment. One participant described the abruptness of having to stop: “When it does happen it generally comes on quick. I don’t get much warning ahead of time. And I have to stop.” Another with severe COPD described,

When I go to the doctor, my wife drops me off and parks the van. And I’ll go into the hospital. Generally just inside the door there’s some wheelchairs sitting around in there. Most times what I’ve done is I’ll grab one of them as a walker. But the last couple times that I’ve been there, there hasn’t been one. So just walking from the front door to the elevator, I have to stop. I get halfway! I don’t have a choice but to stop. Completely. Whatever I’m doing I gotta, I have to stop.

For several participants, everyday actions such as walking across the room or getting up quickly were no longer spontaneous. At one moment they were going across the room or bending over, but in the next moment they had to stop. A participant described having to stop suddenly:

When a thought occurs to me that something’s got to be done, I get up and I go for it. But I’ve hardly gone across the room, when all of a sudden, I can feel my lungs refusing to do the job that they’re supposed to do. My lungs are protesting. Slow down. Quit what you’re doing because I can’t stand it. So very often, if I’m not thinking, I still attempt to do things that physically I’m incapable of doing because of the condition of my lungs.

Another participant, who had been diagnosed 10 years ago with severe COPD, was observed stopping suddenly in the interview. He got up and went about five steps, and then suddenly halted, impeded by the physical body and its need for oxygen. After several moments of standing and pursed lip breathing, he walked the other few steps to the chair, sat down, and again caught his breath. He then stated, “As soon as I go to exert myself, I can’t. I can’t breathe.”

#### Standstill

It is anticipated and understood that we might have to slow down or even stop an activity with strenuous activity, but participants with moderate to very severe lung disease described a very different sort of stopping: There was a suddenness and immediacy of needing to stop. Suddenly, in that moment of time, there was no air and no breath, and they had to stop. The types of exertion that forced them to stop also differed from those experienced by people who are healthy or have a mild disease. Participants had to stop suddenly during activities such as bending, getting up too fast, and walking a few steps too quickly. When their lung and body capacities were unable to achieve such activities, they had to completely stop. They had to stand still, sit down in the middle of a mall, collapse in a chair, or kneel in the middle of a road. Hardly able to keep upright, participants stopped and leaned on whatever was available, such as a table, a fence gate, or a railing. When brought to an abrupt standstill, the participants experienced a suddenness, unexpectedness, and unreliability of the body, as described by this participant:

I remember being in Victoria (coughs) on a conference and I was climbing up and down the hills. And all of a sudden, I just had to stop and hold onto somebody’s gate until I could get my breath rhythm back. And hold on. And work at getting breath into my lungs. Getting air into my lungs.

When brought to a standstill, there was often a perception of being forced to stop. One participant stated when he was short of breath, “All I can do is stop. Whatever I’m doing I just stop. I just can’t do anything else.” Despite his will or intent, his body was no longer mobile; he was no longer moving through the world. He described, “I’m just gasping. I’m down right gasping. I can’t move. I flop in a chair and that’s it. I stay there.”

For participants, this immediate need to stop could be expected and unexpected. When expected, the moments of self-awareness became distinct and sometimes movements were planned. This participant with severe COPD described his stops to get to the front walk:

When I get outside, I’ve already gone up a little short flight of stairs. And I always have to stop outside that door. And lean on that iron railing, until I have recovered. I’m outside. I recover and start walking down the sidewalk there. I typically never get as far as that fencing up there with the building. I stop again. And recuperate. And then I build up a little bit more energy and the second wind and I stop right outside the door and try to get up enough steam.

Another participant with severe asthma described that having to stop in his home was “frustrating,” whereas outside his home “sometimes it gets to be a panicky thing.” He described walking to the store:

It’s two blocks to the corner store over there. I used to be able to walk over there. But when I did that I’d have to stop about five times on the way over there. To catch my breath. I’d just run out of steam. So I got the walker. I can always sit down on it. Without that walker I’m beat. I can’t go anywhere.

## Discussion

Merleau-Ponty (1945/1962) described the phenomenological body as “two distinct layers, that of the habit body and that of the body at this moment” (p. 82). When we move through the world within our phenomenological body, our activities are spontaneous and “movement is not thought about” (Merleau-Ponty, 1945/1962, p. 137). In the taken-for-grantedness of being-in-the-world, there is a “simultaneous patterning of body and world” of our bodies, emotions, and thoughts (Merleau-Ponty, 1945/1962, p. 189). In this manner of being-in-the-world, moments are not reflected upon as objects, and time is seamless between instants and actions. Living in the world and in the body, intentional acts are immediate, spontaneous, and instantaneous, and are not consciously thought about or planned (Merleau-Ponty, 1945/1962).

In contrast to the seamlessness of the habitual body of the moment, these participants frequently had their being-in-the-world interrupted. Their experiences of living with pulmonary disease were not limited to the lungs but extended to the body as a whole and the body-in-the-world. They were no longer able to predictably or habitually move through the world; their immediate future was potentially altered, and their movements were frequently thought about, planned, or impaired. They described moments when their tempo was altered and actions impeded by both breathing and the body. They had to slow down, do less, and at times even stop. In many of these instances, the moment became an object in time and the pre-reflective subjective self was disrupted into reflective objective awareness. The participants were forced to notice and reflect upon the physical body and breathing.

When participants had to stop, it was a demand of the body and a necessity of breathing. Even if and when the embodied, phenomenological self began to move forward again, their body continued to be limited by disease and insufficient air, and the participants often had to stop In these moments, the self was no longer in synchrony and union with the body.

Consistent with other research findings (Boyles et al., 2011; Ek et al., 2011; Habraken et al., 2008), energy limitations restricted activities of participants’ daily lives that would otherwise be taken-for-granted. Participants conserved energy by doing less and incorporated strategies to maintain basic activities, such as showering, dressing, or walking. However, even when doing less and slowing down reduced distressing sensations, symptoms were not completely avoided. All participants described experiences of acute, severe, and distressing episodes of severe shortness of breath.

It is important to be sensitive and empathic to persons with pulmonary disease by attending to their needs to conserve air and energy. For example, nurses might need to limit open-ended questions when patients do not have enough air to speak. It is helpful to teach patients practical strategies to pace themselves, such as going slowly, taking frequent rests, but also clarifying with others the need to do so. Nurses also need to understand the challenges of everyday activities and the burden of symptoms when providing exercise and symptom management education, because there is a significant risk of deconditioning when limiting activities is used to decrease symptoms.

Our daily lives have “living system of meanings” that create immediate recognition and understanding of experiences (Merleau-Ponty, 1945/1962, p. 131). As argued by Carel (2013) and evinced in the lives of the participants, changes brought on by illness affect not only sensory and body experiences but also meanings. For example, a respiratory ailment “not only limits the types of activity in which the ill person can engage, but also reconfigures her understanding of what a range of activity is” (Carel, 2013, p. 347). The change that occurs is in both language and experience: “distances increase, everyday routines take up more time, certain activities have to be forsaken or replaced” (Carel, 2013, p. 353). The experiences of participants in this study elucidate the living systems of meanings of persons with pulmonary disease, and can reshape our perspectives of this disease. This new understanding has implications for the education of patients, the public, and health professionals with respect to COPD and asthma.

With the onset of disease, participants needed to interpret unfamiliar sensations and develop new meanings. Slowing down was an early indicator of disease for several participants, either with COPD or asthma, but it was not interpreted or perceived to be lung disease or illness at that time. Before the diagnosis of pulmonary disease provided a context of illness, experiences of slowing down were not purposefully unreported. Instead, they were attributed to aging, smoking, being out of condition, or other illnesses. These interpretations limit early diagnosis and management, including delay of smoking cessation. It is important that nurses and other health professionals consistently integrate these perceptions into individual health histories and public education strategies to facilitate earlier diagnosis.

Different meanings for terms and distances were perceived and interpreted by participants. For example, some defined “far” as a walk across a room or half of a set of stairs. They described themselves as going fast and needing to slow down, which had different meanings from their friends and family members. In severe to very severe chronic lower pulmonary disease, airflow is restricted and obstructed, which limits minute volume and vital capacity. As such, health care professionals use physiological and functional terms, such as *restricted* or *obstructed*, to identify and describe the limiting effects of reduced airflow on their lungs or breathing. Participants, however, used these terms to describe experiences of living with pulmonary disease rather than their lung volume or airflow. For example, participants with asthma referred to being restricted in choices or actions, such as eating certain foods or attending certain venues; those with COPD spoke of being obstructed from activities, such as golfing or walking with family members.

From a phenomenological perspective, it is important to understand that required capacity is more than the physical air required for breathing: insufficient air determined participants’ capacity for exertions and activities. Therefore, I recommend that both experiential and technical meanings of terms such as capacity, work of breathing, obstruction, and restriction be included when teaching pulmonary pathophysiology and assessment.

It is important to recognize that acute and severe dyspnea, which might be both predictable and unpredictable, are prevalent and distressing symptoms in pulmonary disease. All participants described anxiety, fear, panic, or distress with shortness of breath and with having to stop to get air in. For the participants, the sensation of being unable to breathe was intense, distressing, and frightening, which has been described by participants with COPD or asthma in other qualitative studies (Bailey, 2004; Barnett, 2005; Clancy et al., 2009; Denford, Campbell, Frost, & Greaves, 2013; Fraser et al., 2006; Gardiner et al., 2009). These findings support the recommendation to assess multidimensional aspects of dyspnea, such as intensity, the affective component (including distress), and the impact or burden for the individual (Parshall et al., 2012).

Many of the participants lived a more sedentary life than they had previously or wished to do so at present. They identified two reasons for limiting or restricting their activities: to minimize distressing sensations and insufficient energy. Most participants focused on that which they could no longer do, and several described their struggles with being held back by the symptoms of pulmonary disease and the challenges of breathing. These life changes are associated with social isolation and personal losses (Barnett, 2005; Boyles et al., 2011; Clancy et al., 2009; Ek et al., 2011; Fraser et al., 2006; Gullick & Stainton, 2008; Habraken et al., 2008; Hasson et al., 2008; Jonsdottir, 1998; Williams et al., 2007). Family members, caregivers, and health professionals should be made aware through education that a sedentary lifestyle might be necessitated by the body and is not by choice. This approach would increase sensitivity to the potentially frustrating and distressing limitations. Nurses are also encouraged to develop strategies for patients to maintain activities and engage with others, particularly focusing on individualized activities that are of importance and give meaning.

## Conclusion

The lens of Merleau-Ponty (1945/1962, 1964) provided insight into participants’ experiences with moderate to severe COPD, asthma, or both. The disruption of the habitual, phenomenological body by pulmonary disease was apparent, and the often invisible, yet distressing, experiences of living with moderate to severe COPD or asthma were revealed. Impaired by their inability to breathe or distressed by severe shortness of breath, participants altered their mobility, tried to avoid triggers, slowed their speed, and limited their activities. The findings from this study have extended our understanding of the prevalence of symptoms and distressing experiences of adults living with chronic lower pulmonary illness. Considerations from these findings were described for nursing practice and education.
